# Physical anhedonia in the acute phase of schizophrenia

**DOI:** 10.1186/1744-859X-5-1

**Published:** 2006-01-18

**Authors:** Vassilis P Kontaxakis, Costas T Kollias, Beata J Havaki-Kontaxaki, Maria M Margariti, Sophia S Stamouli, Eleni Petridou, George N Christodoulou

**Affiliations:** 1 Department of Psychiatry, University of Athens, Eginition Hospital, 74 Vas. Sophias Ave, 11528 Athens, Greece; 2 Department of Hygiene and Epidemiology, University of Athens, 75 Micras Asias Ave, 11527, Goudi, Athens, Greece; 3 Hellenic Centre for Mental Health and Research, 58 Notara Ave, 10683 Athens, Greece

## Abstract

**Background:**

The aim of the current study is to investigate the relationship between physical anhedonia and psychopathological parameters, pharmacological parameters or motor side-effects in a sample of inpatients with schizophrenia in an acute episode of their illness.

**Method:**

Eighty one patients with schizophrenia, consecutively admitted, with an acute episode of their illness, at the Eginition Hospital, Department of Psychiatry, University of Athens, during a one-year period were investigated regarding possible relationships between physical anhedonia, social-demographic data and clinical parameters as well as motor side-effects, induced by antipsychotic agents. All patients were assessed using the Chapman Revised Physical Anhedonia Scale (RPAS), the Positive and Negative Syndrome Scale (PANSS), the Rating Scale for Extrapyramidal Side-Effects (EPSE), the Barnes Akathisia Rating Scale (BARS) and the Abnormal Involuntary Movement Scale (AIMS). Simple cross tabulations were initially employed. Subsequently, multiple regression analysis was performed.

**Results:**

Both positive and negative symptoms were associated with physical anhedonia. A positive association between physical anhedonia and the non-paranoid sub-category of schizophrenia was also proved.

**Conclusion:**

According to these results, it seems that in the acute phase of schizophrenia, physical anhedonia may be a contributing factor to patient's psychopathology.

## Background

Anhedonia, a term first used by Ribot [1], describes the lack of interest and the withdrawal from all the usual and pleasant activities. Anhedonia has been described as a schizophrenic symptom by many authors, including Bleuler and Kraepelin [2,3]. Rado [4,5] had suggested that anhedonia is a central, genetically transmitted defect both in overt schizophrenia and in compensated schizotypes. Meehl [6] has integrated Rado's views into a theory of neurological dysfunction in schizophrenia and proposed that anhedonia is an enduring trait, a "cardinal symptom" preceding and possibly causing schizophrenia.

According to Chapman et al [7], there are two types of anhedonia, physical anhedonia and social anhedonia. Physical anhedonia which, usually, precedes the onset of the disease, represents a defect in the ability to experience physical pleasures, such as pleasures of eating, touching e. t. c., while social anhedonia represents a defect in the ability to experience interpersonal pleasure, such as pleasure of being with people, talking e. t. c.

There have been contradictory results regarding the association of anhedonia or its components to clinical parameters (i.e. negative symptoms, positive symptoms, depression) or to drug – treatment. Furthermore, there is a lack of studies regarding the relationship between physical anhedonia and psychopathological parameters in the acute phase of schizophrenia or between physical anhedonia and motor side – effects induced by antipsychotic agents.

The aim of the current study was to investigate the relationship between physical anhedonia and social-demographic, clinical parameters as well as motor side-effects in a sample of inpatients with schizophrenia in the acute phase of their illness.

## Methods

### Subjects

All patients with schizophrenia, consecutively admitted, with an acute episode of their illness, at the Eginition Hospital, Department of Psychiatry, University of Athens, during a one-year period were studied. Written informed consent was obtained from the subjects and their relatives.

The patients' diagnoses were made by two independent psychiatrists of similar level of education and experience according to DSM-IV criteria [8] and were reviewed on the day of discharge, taking into account all information collected during the inpatient period. Patients who presented with any other diagnosis on Axis I of DSM-IV, serious physical illness (especially neurological), substance abuse and mental retardation were excluded from the study.

Eighty one patients were finally diagnosed as suffering from schizophrenia. Fifty were male (62%) and 31 female (38%). Their mean age was 30.95 (± 8.91) years, (age range 17 to 50 years). They had a mean of 12.6 (± 2.7) years of education, a mean duration of illness of 6.9 (± 7.6) years and a mean duration of hospitalisations of 0.4 (± 0.8) years. Most of the patients were single (85%). Patients were divided into the following subcategories: Paranoid type (57%), undifferentiated type (20%), disorganised type (13%), residual type (10%). 27 patients (34%) were for the first time admitted while 54 (66%) had more than one admissions (relapsers).

At the time of assessment, 65 patients (77%) were receiving antipsychotic drugs. Out of a total of 81 patients on antipsychotic drugs, 62% were receiving conventional antipsychotics, 27% used atypical antipsychotics as monotherapy and 12% used conventional plus atypical antipsychotics in combination. Eleven patients (13.6%) were consuming antidepressants, 41 (50.6%) anxiolytics, 2 (2.5%) mood stabilizers and 45 (55.6%) anti-parkinsonian agents.

### Clinical assessments

All patients were assessed using the following scales: the Chapman Revised Physical Anhedonia Scale [RPAS] (9), the Positive and Negative Syndrome Scale (PANSS) [10,11], the Rating Scale for Extrapyramidal Side-Effects (EPSE) [12], the Barnes Akathisia Rating Scale (BARS) [13] and the Abnormal Involuntary Movement Scale (AIMS) [14,15]. The severity of depression was estimated using the depression cluster score of the PANSS (items G1+G2+G3+G6) [16,17].

Means and standard deviations of the main variables are shown in table 1.

**Table 1:** Mean patients' scores

Scale	Mean	(SD)
PAS	19.10	(± 7.38)
PANSS – Positive symptoms	18.47	(± 6.61)
PANSS – Negative symptoms	20.20	(± 8.09)
PANSS – General psychopathology symptoms	39.27	(± 11.30)
PANSS – Depression	9.91	(± 2.94)
EPSE	0.76	(± 0.58)
BARS	0.30	(± 0.53)
AIMS	0.12	(± 0.30)

PAS: Physical Anhedonia Scale, PANSS: Positive and Negative Syndrome Scale, EPSE: Rating Scale for Extrapyramidal Side-Effects, BARS: the Barnes Akathisia Rating Scale, AIMS: Abnormal Involuntary Movement Scale

Subjects were assessed during the first week of their hospitalisation by three independent psychiatrists-raters. The first rater assessed the patients using the RPAS and the AIMS, the second using the PANSS and the EPSE and the third using the BARS. Information from the patient's history, concerning social-demographic and clinical parameters was recorded in a pre-coded interview form. The antipsychotic agents dosage was estimated in chlorpromazine equivalents [18,19].

### Statistical analyses

The SPSS 8.0 was used for the statistical analysis. Since there is no cut-off point for schizophrenia, dividing anhedonic subjects from non-anhedonic ones, physical anhedonia scores were divided in thirtiles according to the ratings in rPAS (1^st ^thirtile: <15 and <14, 2^nd ^thirtile: 16–22 and 15–21, 3^rd ^thirtile: >23 and >22 for men and women respectively). Then, possible correlations were explored between: physical anhedonia and social-demographic parameters (i.e. sex, age, family status e.t.c.), clinical parameters (i.e. diagnostic sub-category) and psychopathological parameters derived from the aforementioned scales and their subscales used. Simple cross tabulations were initially employed. Of all the parameters cross-tabulated with physical anhedonia, statistically significant differences between subjects with lower anhedonia scores and subjects with higher anhedonia scores were found only for the PANSS positive sub-scale score, the PANSS negative sub-scale score and the dianostic sub-category parameter. Subsequently, multiple regression analysis was performed, using as predictor core model the following variables: gender, age, family status, diagnosis and years of education and diagnostic sub-category. Alternative introduced clinical standard variables to the core model were the PANSS positive sub-scale score and the PANSS negative sub-scale score. The physical anhedonia score was the dependent variable.

## Results

Tables 2 shows the distribution of the sample crossclassified by sociodemographic, clinical variables and the physical anhedonia score thirtiles. The severity of physical anhedonia was significantly related to the diagnostic sub-category of non-paranoid schizophrenia, to the positive symptoms score and to the negative symptoms score of the PANSS.

**Table 2:** Distribution of 81 patients with schizophrenia by sociodemographic and clinical variables and the percentages of the physical anhedonia score calculated for each gender

Variable	physical anhedonia score thirtiles						p=
	1^st^	(%)	2^nd^	(%)	3^rd^	(%)	
Gender							
Male	17	34.0	15	30.0	18	36.0	0.64
Female	10	32.3	11	35.6	10	32.3	
Age (years)							
< 25 years	10	43.5	10	43.5	3	13.0	0.09
25–34	9	29.0	8	25.8	14	45.2	
>35	8	29.6	8	29.6	11	40.8	
Education (years)							
≤ 12	13	28.9	15	33.3	17	37.8	0.35
>13	14	38.8	11	30.6	11	30.6	
Family status							
Unmarried	19	27.5	26	37.7	24	34.8	0.12
Other	8	66.7	0	0.0	4	33.3	
Diagnostic sub-category							
Paranoid	22	47.8	13	28.3	11	23.9	0.002*
Other	5	14.3	13	37.1	17	48.6	
PANSS-positive subscale score							
≤ 19	20	42.6	15	31.9	12	25.5	0.02*
>20	7	20.6	11	32.3	16	47.1	
PANSS-negative subscale score							
≤ 19	17	40.5	15	35.7	10	23.8	0.04*
>20	10	25.6	11	28.2	18	46.2	

*statistically significant difference, Chi – square tests

Table 3 shows the associations of physical anhedonia scores with the core model variables and the multiple regression analysis results. There were statistically significant associations of physical anhedonia scores with alternative clinical variables which were introduced to the core model. Single patients tended to have higher scores of physical anhedonia than others (p = 0.05). Older patients tended to score higher on physical anhedonia (p = 0.05). Patients with paranoid schizophrenia had lower scores of physical anhedonia than non-paranoid patients with schizophrenia (p = 0.004). Both positive symptoms score and negative symptoms score were positive predictors of physical anhedonia (P = 0.03 and P = 0.01, respectively).

**Table 3:** Multiple regression-derived partial regression coefficients (b), standard errors (SE) and corresponding p-values for prediction of physical anhedonia from core model variables and clinical standard variables

Variable	Category	B	SE	p=
*Core model*				
Gender				
	Male			
	Female	-0.41	1.63	0.81
Age (years)				
	<25			
	25–34			
	>35	1.99	1.01	0.05
Education (years)				
	≤ 12 years			
	>13	-2.13	1.55	0.17
Family Status				
	Unmarried			
	Other	-4.69	2.31	0.05
Diagnostic sub-category				
	Paranoid			
	Other	4.63	1.57	0.004 *
*Alternative introduced variables to the core model*				
*Model 1*				
PANSS-positive subscale score	≤ 19			
	>20	3.34	1.54	0.03 *
*Model 2*				
PANSS-negative subscale score	≤ 19			
	>20	3.89	1.53	0.01 *

* statistically significant difference

## Discussion

This is the first study searching simultaneously for possible association between physical anhedonia and positive symptoms, negative symptoms or general psychopathology symptoms as well as motor side effects induced by antipsychotic agents in inpatients with schizophrenia in the acute phase of their illness.

Starting with, we should mention several limitations of our study. First, we used a mixed population of patients with schizophrenia regarding their medication status. Second, there was a lack of a specific scale measuring depression in schizophrenia. Third, we studied patients in the acute phase of their illness. Hence, it was possible that positive symptoms may dominate and overlap the clinical manifestation of the disease.

According to our results, the severity of physical anhedonia was associated with the severity of both positive and negative symptoms. Also, a positive association between physical anhedonia and the sub-category of non-paranoid schizophrenia was presented. However, the severity of physical anhedonia was found to be independent to depression, to general psychopathology symptoms or motor side-effects induced by antipsychotic agents.

Regarding the relationship of physical anhedonia and negative symptoms the results are in line with those by Loas et al [20] and Kirkpatrick et al [21] who have demonstrated that physical anhedonia and deficit symptoms, which are described as enduring negative symptoms, were significantly related. Yet, Herbener et al [22] found that the PAS score of patients with schizophrenia was significantly correlated to negative symptoms, estimated by the SADS structured interview, at the 4.5 year follow up assessment. However, contrary to our results, Loas et al [23] found that physical anhedonia in chronic patients with schizophrenia was not significantly related to negative symptoms, estimated by both the PANSS and the BPRS. Other studies, as well, have reported an absence of significant correlation between physical anhedonia and negative symptoms as measured by the Positive and Negative Symptoms Scale (PANSS), the Brief Psychiatric Rating Scale (BPRS), or the SANS [24-26].

Regarding the relationship of physical anhedonia and positive symptoms, contrary to our results, Herbener et al [22] found that the PAS score of patients with schizophrenia was not correlated to florid psychotic symptoms, estimated by the SADS, over a 10 – year follow up period. Also, Loas et al [23] found that physical anhedonia in chronic patients with schizophrenia was not significantly related to positive symptoms, estimated by both the PANSS and the BPRS.

Regarding the relationship of physical anhedonia and the non-paranoid sub-type of schizophrenia, contrary to our results, Schunck et al [27] did not find a correlation between the PAS score and the schizophrenia sub-type in a sample of out-patients with schizophrenia.

The observation that physical anhedonia is independent of depression in schizophrenia seems to be consistent with the findings of Herbener et al [22]. They used a sub-scale of the SADS to estimate depression. Also, Loas et al [23] have found that physical anhedonia in chronic patients with schizophrenia was not significantly related to depression, estimated by the Beck Depression Inventory.

Regarding the relationship of physical anhedonia and general psychopatology symptoms, similar to our results are the findings of Loas et al [23] who have found that physical anhedonia in chronic patients with schizophrenia was not significantly related to general psychopathology symptoms, estimated by both the PANSS and the BPRS.

We did not find any studies exploring the the relationship between physical anhedonia and motor side effects induced by antipsychotic agents.

## Conclusion

According to our results, it seems that in the acute phase of schizophrenia, physical anhedonia may be a component of patient's psychopathology. Further studies to elucidate the nature of physical anhedonia and its relationship to various phases of the schizophrenic disorder are needed.
